# Cangrelor in Patients Undergoing Percutaneous Coronary Intervention After Out-of-Hospital Cardiac Arrest

**DOI:** 10.3390/jcm14010076

**Published:** 2024-12-27

**Authors:** Marco Ferlini, Luca Raone, Sara Bendotti, Alessia Currao, Roberto Primi, Andrea Bongiorno, Cristian Fava, Laura Dall’Oglio, Marianna Adamo, Daniele Ghiraldin, Marcello Marino, Cinzia Dossena, Andrea Baldo, Diego Maffeo, Vilma Kajana, Silvia Affinito, Enrico Baldi, Leonardo De Luca, Simone Savastano

**Affiliations:** 1 Division of Cardiology, Fondazione IRCCS Policlinico San Matteo, 27100 Pavia, Italy; marco.ferlini@gmail.com (M.F.); s.bendotti@smatteo.pv.it (S.B.); a.currao@smatteo.pv.it (A.C.); r.primi@smatteo.pv.it (R.P.); andrea.bongiorno.93@gmail.com (A.B.); enrico.baldi@unipv.it (E.B.); leo.deluca@libero.it (L.D.L.); 2 Department of Molecular Medicine, University of Pavia, 27100 Pavia, Italy; 3 Cardiac Arrest and Resuscitation Science Research Team (RESTART), Fondazione IRCCS Policlinico San Matteo, 27100 Pavia, Italy; 4 Department of Internal Medicine and Medical Therapy, University of Pavia, 27100 Pavia, Italy; 5 Department of Public Health, Experimental and Forensic Medicine, University of Pavia, 27100 Pavia, Italy; 6 Division of Cardiology, Carlo Poma Hospital, 46100 Mantua, Italy; cristian.fava@asst-mantova.it (C.F.); laura.dalloglio@asst-mantova.it (L.D.); 7 Cardiothoracic Department, ASST “Spedali Civili”, 25121 Brescia, Italy; mariannaadamo@hotmail.com (M.A.);; 8 Division of Cardiology, Maggiore Hospital, 26013 Crema, Italy; marcello.marino83@hotmail.it (M.M.); cinzia.dossena@asst-crema.it (C.D.); 9 Division of Cardiology, Sant’Anna Hospital, 22100 Como, Italy; andreabaldo25@gmail.com; 10 Interventional Cardiology Unit, Fondazione Poliambulanza Hospital Institute, 25124 Brescia, Italy; diego.maffeo@poliambulanza.it; 11 Division of Cardiology, Clinical Institute Humanitas, 21053 Castellanza, Italy; vilmakajana@yahoo.it; 12 Division of Cardiology, Hospital ASST Ovest Milanese, 200025 Legnano, Italy; silviaaffinito@yahoo.it

**Keywords:** coronary artery disease, cangrelor, out-of-hospital cardiac arrest, platelet aggregation inhibitors, invasive coronary angiography, percutaneous coronary intervention

## Abstract

**Background**: Cangrelor provides rapid platelet inhibition, making it a potential option for out-of-hospital cardiac arrest (OHCA) survivors undergoing percutaneous coronary intervention (PCI). However, clinical data on its use after OHCA are limited. This study investigates in-hospital outcomes of cangrelor use in this population. **Methods**: We conducted a prospective, observational study involving OHCA patients from the Lombardia CARe Registry (January 2015–December 2022) who underwent PCI in seven centers in Northern Italy. Propensity score (PS) matching compared patients who received cangrelor to those who did not. Logistic regression tested associations between cangrelor and discharge outcomes. **Results**: Of 612 OHCA patients admitted, 414 (67.4%) underwent PCI with known antithrombotic therapy, of whom 34 (8.2%) received cangrelor. Radial access was more common in the cangrelor group, which also had a higher troponin peak and a final TIMI flow grade of 3. Survival at discharge was 82.4% in the cangrelor group, compared to 65.3% in the no-cangrelor group (*p* = 0.043). Univariable logistic regression showed that cangrelor use was associated with higher survival at discharge (OR 2.5; 95% CI: 1.1–6.1, *p* = 0.049). After multiple PS matchings, cangrelor remained associated with better survival (OR 2.07; 95% CI: 1.16–2.98). Major bleeding rates were higher in the cangrelor group, even after adjusting for baseline bleeding risk (OR: 7.0; 95% CI: 2.9–17.0; *p* < 0.001). **Conclusions**: In OHCA patients undergoing PCI, cangrelor use was linked to improved in-hospital survival but higher major bleeding, suggesting a potential net clinical benefit.

## 1. Introduction

Acute coronary syndrome (ACS) is the most common cause of out-of-hospital cardiac arrest (OHCA) [1]. Emergent invasive coronary angiography (ICA) and percutaneous coronary intervention (PCI) have been shown to improve outcomes in unstable patients with non-persistent ST segment elevation and in those presenting persistent ST segment elevation [2]. 

Comatose survivors of OHCA undergoing PCI are a challenging population being at higher risk of thrombotic and bleeding complications. A delayed onset of pharmacodynamic effects has been reported in OHCA with both clopidogrel and the more potent oral P2Y12 inhibitors, prasugrel and ticagrelor; the impaired inhibition of platelet aggregation with oral drugs can be caused by gastric paresis, reduced gastric/intestinal perfusion due to reduced cardiac output, hypothermia itself, and increased systemic inflammation related to resuscitation [3].

Cangrelor is an intravenous adenosine triphosphate analog with high selectivity for P2Y12 receptors that does not require the cytochrome 450 or other enzymatic activation: after the bolus dose administration, it achieves a full and sustained platelet inhibition within few minutes; furthermore, for its reversible binding to receptors, a complete platelet function recovery can be achieved within 1 h after the infusion ends [4]. Based on these pharmacodynamics characteristics, cangrelor may overcome the limitations of oral P2Y12 inhibitors in acute settings, particularly if their delayed effect can be highly expected. 

In comatose survivors of OHCA undergoing PCI, cangrelor use has been shown to induce faster and more sustained inhibition of platelet aggregation compared to all three P2Y12 inhibitors without a significant increase in bleeding events [5,6,7,8]. However, most available data have been obtained in a small population and on pharmacodynamic endpoints. 

We aim to investigate the effect of cangrelor on clinical outcomes in a real-world cohort of patients with OHCA as a complication of ACS undergoing PCI.

## 2. Materials and Methods

### 2.1. Study Design and Patient Selection

This is a multicenter, retrospective analysis of a prospective data collection, including all consecutive patients with OHCA enrolled in the Lombardia CARe Registry (ClinicalTrials.gov ID NCT03197142) from 1 January 2015 to 31 December 2022 who underwent ICA in 7 centers in Northern Italy (Fondazione IRCCS Policlinico San Matteo in Pavia, ASST Spedali Civili in Brescia, Sant’Anna Hospital in Como, Maggiore Hospital in Crema, Hospital ASST Ovest Milanese in Legnano, Fondazione Poliambulanza Institute in Brescia, and Clinical Institute Humanitas in Castellanza).

### 2.2. Study Outcomes

The primary endpoint of this analysis was defined as the rate of patient survival at hospital discharge. Additionally, the incidence of major bleeding events during hospitalization was assessed as the safety endpoint.

### 2.3. Cardiac Arrest Registry

The Lombardia Cardiac Arrest Registry (Lombardia CARe—NCT03197142) is a multicenter longitudinal prospective Utstein-based registry enrolling all the OHCA cases for which the Emergency Medical System (EMS) is alerted in the Province of Pavia since 1 January 2015 with the addition of the provinces of Lodi, Cremona, and Mantua from 1 January 2019, the Province of Varese from 1 January 2020, and the provinces of Como and Brescia from 1 January 2021. All the data were collected according to the Utstein 2014 recommendations [9] The Lombardia CARe was approved by the ethical committee of the IRCSS Policlinico San Matteo Foundation (proc. 20140028219) and by all other centers’ ethical committees. An informed consent form was signed by all the patients discharged alive.

### 2.4. Procedures and Definitions

After the return of spontaneous circulation (ROSC), ICA was performed in accordance with current European Society of Cardiology (ESC) guidelines [10] in cases of ST-segment elevation myocardial infarction or hemodynamically or electrically unstable non-ST-segment elevation myocardial infarction. The decision to proceed with ICA, including its timing, was determined through consensus between clinical and interventional cardiologists and intensivists involved in patient evaluation.

The choice of vascular access, the type and number of stents, and the peri-procedural antithrombotic therapy were left to the discretion of the interventional cardiologists. Bleeding events were classified according to the Bleeding Academic Research Consortium (BARC) scale [11], which ranges from 0 (no bleeding) to 5 (probable or definite fatal bleeding). Major bleeding was defined as BARC grades 3 to 5.

High bleeding risk (HBR) was defined by the presence of at least two of the following criteria: renal impairment (estimated glomerular filtration rate [eGFR] < 30 mL/min), liver disease, active cancer, low platelet count, history of previous bleeding or blood transfusion, ongoing anticoagulation therapy, use of NSAIDs or steroids, or recent surgery or trauma [12].

### 2.5. Antithrombotic Therapy

As previously mentioned, any decision about antithrombotic therapy was left to the discretion of the interventional cardiologist that performed the PCI. However, all patients received intravenous aspirin 250 mg at first medical contact or if not, before the cannulation of the target vessel with the guiding catheter. After ICA and confirmation of the need to proceed with PCI, unfractionated heparin was dosed at 70 units/kg, with a target ACT > 300″ that was checked every 30 min. 

Cangrelor was administered according to the label, as a bolus (30 μg/kg), followed by continuous infusion of 4 μg/kg/min for a median time of 2 h. In patients receiving cangrelor, the transition to oral P2Y12 inhibitors was managed as follows: ticagrelor (180 mg loading dose) was administered within 30 min after the end of the infusion, while clopidogrel (600 mg loading dose) and prasugrel (60 mg loading dose) were given immediately at the end of the infusion. For patients not treated with cangrelor, oral P2Y12 inhibitors were administered with a loading dose at the end of the interventional procedure. The use of Glycoprotein IIb/IIIa inhibitors (GPIs) was restricted to bailout situations only.

### 2.6. Data Managing

All data regarding the pre-hospital treatment were automatically obtained from the regional EMS data warehouse and filed in an electronic online database (REDCap 14.0.15—© 2024 Vanderbilt University). For each province, one or more EMS investigators were asked to correct and verify the pre-hospital data. Clinical investigators for each hospital were responsible for completing data about patient’s in-hospital stay. The Lombardia CARe Study Management Team was responsible for the quality control of data.

### 2.7. Statistical Analysis

Categorical variables were described as a number and percentage and compared with the chi-squared test or Fisher’s exact test depending on the expected frequencies. Continuous variables were described as the mean ± standard deviation and compared with the *t*-test or described as the median and interquartile range (IQR) and compared with the Mann–Whitney test and according to their normal distribution tested with the Shapiro–Wilk test. All the variables that differed significantly between patients treated with cangrelor and patients in whom cangrelor was not administered were included in a multivariable logistic model for cangrelor administration. Model goodness of fit was assessed with Pearson’s test. The sensitivity and specificity of the model were also computed. From the resulting coefficients, a propensity score was calculated. Patients were randomly matched according to their propensity score to generate random samples. The number of samples needed was established according to the convergence of the median chi-squared test. The goodness of the propensity score matching was evaluated in terms of balance of the baseline characteristics comparing the propensity distribution graph in the unmatched population and matched population and the Kolmogorov–Smirnov test [13]. For each sample, considering only matched patients, a chi-squared test and logistic regression were performed to test the association between cangrelor administration and patient survival at discharge. The median chi-squared test and the overall odds ratio (OR) derived from each sample were taken into account to confirm the association between cangrelor administration and the primary efficacy endpoint. A chi-squared test and logistic regression were performed to assess the association between cangrelor and the primary safety endpoint. Statistical analysis was performed with STATA 17 (StataCorp. 2021. Stata Statistical Software: Release 17. College Station, TX, USA: StataCorp LLC.). A two-sided *p*-value of <0.05 was considered statistically significant.

## 3. Results

### 3.1. Baseline Characteristics

**Figure 1:**
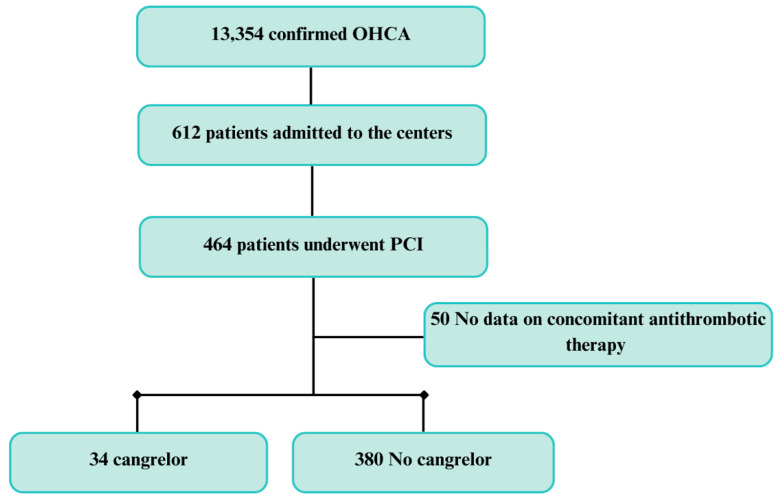
Study flow-chart. OHCA: out-of-hospital cardiac arrest; ICA: invasive coronary angiography.

During the study period, a total of 13,354 OHCAs were treated by emergency medical services (EMSs) within the area covered by the Lombardia CARe registry. Of these, 612 patients were admitted to the seven centers involved in this study: 464 (60.3%) underwent a PCI, but 50 of them were excluded for missing information about antithrombotic therapy; therefore, 414 patients were considered as the population of the present analysis. Cangrelor (study group) was administered in 34 cases (8.2%), while the remaining 380 were considered as the control group (Figure 1). 

Table 1 summarizes the baseline characteristics of the study population. No significant differences were found between the two groups regarding median age [64.0 (55.0–72.0) years] or gender distribution (77.5% male). Patients in the cangrelor group were more frequently treated with vasopressors (64.7% vs. 36.3%; *p* = 0.004), had higher rates of radial access (61.8% vs. 42.9%), and more frequently underwent PCI of the left main coronary artery (14.7% vs. 4.7%; *p* = 0.045). Additionally, they exhibited a higher peak troponin level [75,806.5 (20,014.0–110,936.0) ng/L vs. 11,718.0 (1836.5–61,289.5) ng/L; *p* = 0.005] and were more likely to achieve a final TIMI 3 flow (91.2%. vs. 64.2%; *p* = 0.024). No significant differences were observed in OHCA characteristics, including location, presence of witnesses, presenting rhythm, EMS arrival time, and resuscitation duration. Matched cohort characteristics are detailed in Supplementary Table S1 and S2.

### 3.2. Clinical Outcomes

A higher proportion of patients in the cangrelor group were alive at hospital discharge compared to the control [82.4% vs. 65.3% (chi-squared: 4.1015; *p*-value: 0.043)]. According to univariable logistic regression, use of cangrelor was associated with higher odds of survival at discharge (OR 2.5; 95% CI: 1.1–6.1, *p*-value = 0.049).

**Figure 2:**
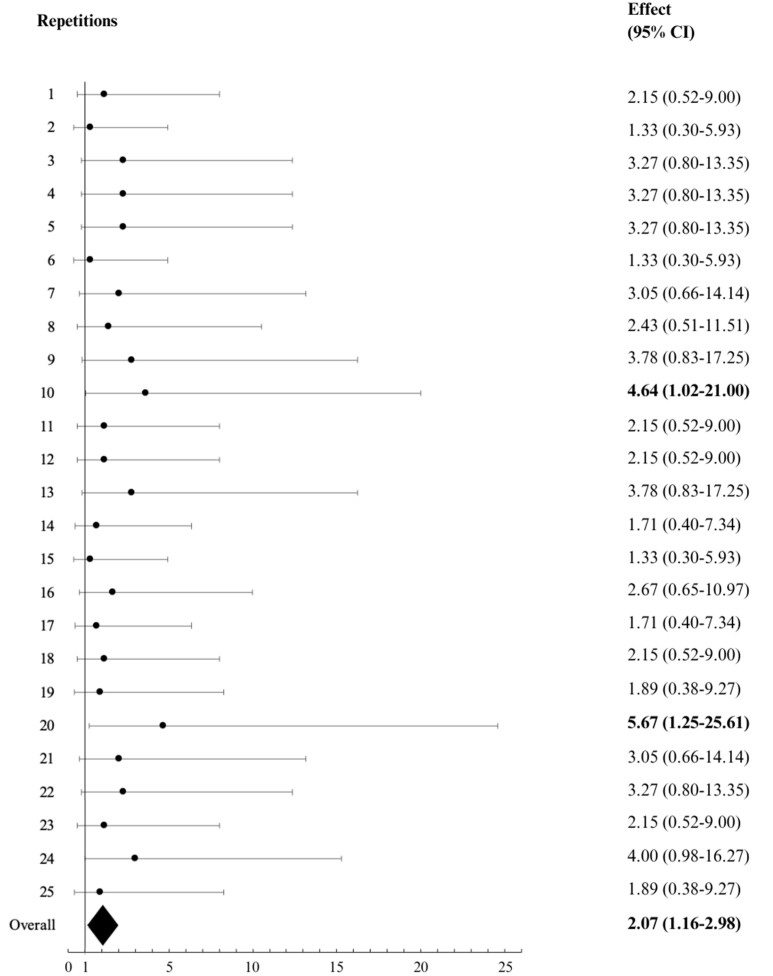
Forest plot displaying the effect of cangrelor administration on in-hospital survival derived from 25 propensity-score-matched samples. Black circles represent the odds ratio (OR), and horizontal lines represent the 95% confidence interval (CI) of each one of the 25 matched samples. The overall effect is displayed at the bottom (black diamond). The convergence of the median chi-squared test displayed in Supplementary Figure S1 confirms the robustness of the matching methodology. The bold numbers indicate the significant pairs.

Table 2 shows the resulting coefficients used for the computation of the propensity score and obtained from a multivariable logistic regression model for the probability of receiving cangrelor run with all the significantly different variables between the cangrelor group and the no-cangrelor group. The area under the ROC curve (AUC) of the model was 0.82. Patients were randomly matched according to their propensity score, and 25 random samples with 20 patients each (cangrelor and no-cangrelor group) were obtained (Table 3) as indicated by the median chi-squared test convergence (Supplementary Figure S1). Figure 2 illustrates the resulting ORs for survival at discharge, with an overall OR of 2.07 (95% CI: 1.16–2.98), confirming a significant survival advantage associated with cangrelor use. However, this survival benefit was counterbalanced by a higher incidence of major bleeding in the cangrelor group compared to the control group (33.0% vs. 7.5%; OR: 6.2; 95% CI: 2.6–14.8; *p*-value <0.001). Even after adjusting for baseline HBR characteristics, the risk of major bleeding associated with cangrelor remained significantly elevated (adjusted OR: 7.0; 95% CI: 2.9–17.0; *p*-value < 0.001).

## 4. Discussion

The main findings of the present study enrolling patients with OHCA undergoing PCI can be summarized as follows: the use of cangrelor was low but associated with a significant improvement in survival at hospital discharge, despite a higher rate of major bleeding events.

An adequate peri-procedural inhibition of platelet aggregation is essential in patients undergoing PCI to reduce adverse thrombotic events; in comatose survivors of OHCA, the delayed effect of oral antiplatelet drugs through multiple mechanisms (sedation, nausea, vomiting, shock condition, endotracheal intubation, and hypothermia) contribute to limiting the efficacy of oral antiplatelet agents that has been shown to be overcome with the use of the intravenous cangrelor [3,5,6].

In our analysis, cangrelor was used in a small number of patients, below 10%. Although a comparison with previous data is challenging, as results can be different based on the duration of observations, geographical characteristics, and number of centers involved, in a real-world experience of patients undergoing PCI, cangrelor was administered to 10–15% of cases [8,14,15,16]. In a study of the Swedish Coronary Angiography and Angioplasty Registry (SCAAR), cangrelor was used in 16% of patients with STEMI treated with primary PCI, and of these, about 30% presented with cardiac arrest. Similar to our population, in cardiogenic shock and after cardiopulmonary resuscitation, 143 patients undergoing PCI received cangrelor over two years at three hospitals, although in this case the rate of administration in the overall population was not available [17]. 

The findings of this study underscore the clinical relevance of cangrelor in high-risk PCI settings. The observed improvement in in-hospital survival (OR: 2.07; 95% CI: 1.16–2.98) highlights the efficacy of cangrelor’s rapid and potent platelet inhibition, which may provide a procedural advantage by optimizing reperfusion, as suggested by the higher rate of final TIMI flow grade 3 achieved in the cangrelor group. However, the significantly increased incidence of major bleeding (OR: 7.0; 95% CI: 2.9–17.0) underscores the importance of careful patient selection and thorough risk stratification to balance the benefits and risks of its use. Most previous studies including patients with OHCA focused on pharmacodynamic endpoints; therefore, their sample sizes were too small to draw clinical conclusions. 

In the CHAMPION PHOENIX [18], compared to clopidogrel in patients undergoing PCI naive for P2Y12 inhibitors, cangrelor significantly reduced the co-primary endpoint [death, MI, ischemia driven revascularization (IDR), or stent thrombosis (ST)] at 48 h; however, as a single component, there was no difference in mortality or in IDR, while MI was significantly reduced by cangrelor as much as ST, which was reduced by 38% in the cangrelor arm. In the CHAMPION PHOENIX, patients with STEMI were less than 20% of the whole population, with a risk profile that can be considered low compared to our population. In patients presenting with cardiogenic shock (CS) and after cardiopulmonary resuscitation, cangrelor-treated patients experienced slightly lower mortality at both 30 days and 1 year, without statistical significance. Similar to our results, in the previous study, cangrelor use was associated to a significantly better final TIMI flow, which is known to improve outcome [19,20,21]. A further explanation of our short-term mortality reduction could be found in ST reduction, although we did not collect this endpoint. The occurrence of ST is a rare but potential catastrophic complication, being associated with death or MI in more than 50% of cases; the risk of ST is particularly increased in patients undergoing PCI for ACS, in those with diabetes, and in complex coronary anatomy such as bifurcation, left main, and in case of multiple stent implantation [22]. In TIMI 38 and PLATO, the use of prasugrel and ticagrelor, respectively [23,24], significantly reduced ST compared to their control arm, but not in the first 24–48 h. In the CHAMPION PHOENIX, ST was the key secondary endpoint, and according to study definition, an intraprocedural ST (IPST) was any angiographically new or worsening thrombus occurring during PCI and related to stent implantation; although ranging from 0.5 to 0.7%, the IPST was a strong independent predictor of short- and long-term mortality and of MI, and an independent predictor of ARC-definite ST at 48 h and at 30 days. The immediate platelet inhibition provided by cangrelor significantly reduced IPST by 35% irrespective of clinical setting [25].

Finally, we found an increased risk of major bleeding associated with cangrelor use, even after adjusting for high bleeding risk characteristics. OHCA patients are at an elevated risk of both thrombotic and hemorrhagic events due to several factors. Thrombosis risk is heightened by targeted temperature management and delayed absorption of antiplatelet agents from hypotension and cardiogenic shock; conversely, bleeding risk is increased by femoral access, cardiopulmonary resuscitation maneuvers, and MCS. Additional factors such as post-resuscitation syndrome and impaired renal or hepatic function further complicate this balance, creating a uniquely delicate risk profile in these patients. [3,26,27,28]. 

In a retrospective study including resuscitated patients with ACS, the use of cangrelor did not increase major bleeding both if adjudicated with the TIMI or the BARC grade [7], similarly to the results of the matched-pair analysis with oral P2Y12 inhibition from the IABP-SHOCK II trial where authors reported bleeding events with the GUSTO grade [29]. In our population, the rate of major bleeding in the cangrelor group compared to the two previous studies, which could only be explained for the second one based on the more restrictive GUSTO criteria. In the CHAMPION PHOENIX, bleedings were significantly higher in the cangrelor group only using the ACUITY definition grade, but not with the GUSTO (used for the primary safety analysis) and BARC [15]. However, a clinical benefit despite an increase in bleeding risk has already been reported in different trials investigating potent antiplatelet agents: use of prasugrel or ticagrelor compared to clopidogrel, respectively, significantly increased the risk of major non-CABG-related bleeding [30,31]. Despite the increased bleeding risk, cangrelor may offer a net clinical benefit by potentially reducing periprocedural thrombotic events and leading to a higher incidence of final TIMI flow grade 3 in treated patients. Nevertheless, larger studies are needed to confirm this hypothesis and further define the risk–benefit profile of cangrelor in this high-risk population, and, on this point, it is probably worthwhile for future studies to also explore the management of patients with atrial fibrillation requiring anticoagulation, who represent a significant proportion of patients undergoing PCI [32].

This study has some limitations that need to be mentioned. The primary limitation lies in the observational/retrospective design and the small sample size. While the methodology, including the use of the median chi-squared test convergence, ensures statistical robustness, the small cohort size may limit the generalizability and extrapolative capacity of the findings. Future studies involving larger populations are essential to confirm these results and establish broader applicability. Second, the endpoints were self-reported by investigators and not adjudicated by a clinical event committee, which could be crucial in the case of bleeding events. Third, for the retrospective design, some information such as minor bleeding and further detailed characteristics was missing. Finally, our results cannot be extrapolated as an indication of real-world practice within different Italian regions or be representative of different healthcare systems and geographical areas.

## 5. Conclusions

In real-world patients with OHCA undergoing PCI, the use of cangrelor was associated with improved in-hospital survival despite an increase in bleeding risk. 

## Figures and Tables

**Table 1:** Baseline patient characteristics.

Clinical Characteristics	Cangrelor N = 34	No Cangrelor N = 380	Overall N = 414	*p* Value
Age (years)	64.0 (55.0–73.0)	64.5 (57.0–72.0)	64.0 (55.0–72.0)	1.000
Male	23 (67.6%)	298 (78.4%)	321 (77.5%)	0.15
BMI (kg/m^2^)	25.0 (24.2–27.0)	26.0 (24.2–28.0)	26.0 (24.2–27.8)	0.32
Hypertension	21 (61.8%)	254 (66.8%)	275 (66.4%)	0.56
Diabetes	5 (14.7%)	63 (16.6%)	68 (16.4%)	0.67
Hypercholesterolemia	12 (35.3%)	169 (44.5%)	181 (43.7%)	0.50
Smoke	12 (35.3%)	103 (27.1%)	115 (27.8%)	0.17
Previous AMI	1 (2.9%)	68 (17.9%)	69 (16.7%)	0.16
High bleeding risk *	6 (17.6%)	85 (22.4%)	91 (22.0%)	0.49
PRECISE-DAPT score (n)	18.0 (11.0–29.0)	21.0 (14.0–31.5)	21.0 (13.0–31.0)	0.23
Peak high sensitivity troponin I during index hospitalization (ng/L)	75,806.5 (20,014.0–110,936.0)	11,718.0 (1836.5–61,289.5)	13,479.0 (1900.0–76,050.0)	0.005
ECG diagnostic for STEMI	15 (44.1%)	151 (39.7%)	166 (40.1%)	0.13
Procedural characteristics				
Femoral access site for PCI (n)	11 (32.4%)	212 (55.8%)	223 (53.9%)	0.002
Pharmacological or mechanical hemodynamic support (n)	147 (38.7%)	23 (67.6%)	160 (38.6%)	0.003
Number of stents implanted (n)	1.0 (1.0–2.0)	1.0 (0.0–2.0)	1.0 (0.0–2.0)	<0.001
Total length of stent implanted (mm)	38.0 (28.0–48.0)	33.0 (22.0–48-0)	33.0 (23.0–48.0)	0.22
LM PCI (n)	5 (14.7%)	18 (4.7%)	23 (5.6%)	0.045
GP IIb/IIIa receptor inhibitors used during PCI	3 (8.8%)	123 (32.3%)	126 (30.4%)	0.015
P2Y12 receptor inhibitors used during PCI				<0.001
Clopidogrel	9 (26.5%)	86 (22.6%)	95 (22.9%)	
Ticagrelor	25 (73.5%)	161 (42.4%)	186 (44.9%)	
Prasugrel	0 (0.0%)	5 (1.3%)	5 (1.2%)	
Duration of cangrelor infusion (h)	2.0 (2.0–2.9)	/		
Final TIMI flow 3 (n)	31 (91.2%)	244 (64.2%)	275 (66.4%)	0.024
OHCA characteristics				
Shockable rhythm at presentation	31 (91.2%)	299 (78.7%)	330 (79.7%)	0.083
Cardiac arrest time (min)	21.6 (6.0–33.2)	22.0 (10.0–38.0)	22.0 (10.0–37.0)	0.55
N° of delivered shocks (n)	2.0 (1.0–5.0)	2.0 (1.0–4.0)	2.0 (1.0–4.0)	0.52

BMI: body mass index; AMI: acute myocardial infarction; STEMI: ST-elevation myocardial infarction; PCI: percutaneous coronary intervention; LM: left main; TIMI: thrombolysis in myocardial infarction; OHCA: out-of-hospital cardiac arrest. * High bleeding risk was defined as the presence of two of the following clinical conditions: renal impairment (eGFR < 30 mL/min), liver disease, active cancer, low platelet count, previous bleeding/blood transfusion, anticoagulation therapy, NSAIDs/steroid therapy, and recent surgery trauma.

**Table 2:** Multivariable logistic regression for the probability of receiving cangrelor.

Variable	Coefficient	OR	95% CI	*p*-Value
Pharmacological hemodynamic support	1.6	4.8	1.4–16.6	0.014
Access site for PCI	−1.3	0.3	0.1–0.8	0.024
LM PCI	1.3	3.8	0.8–18.7	0.096
Previous anticoagulant therapy	−0.6	0.6	0.03–9.4	0.694
ASA LD during PCI	0.7	2.0	0.6–6.9	0.294
WBC on admission	0.0	1.0	1.0–1.0	0.297
Peak high sensitivity troponin I during index hospitalization	0.0	1.4	1.1–1.9	0.026
Number of stents implanted	0.3	1.3	0.7–2.3	0.452
Final TIMI flow 3	0.5	1.5	0.1–16.2	0.722

PCI: percutaneous coronary intervention; LM: left main; TIMI: thrombolysis in myocardial infarction; LD: loading dose; WBC: white blood cell.

**Table 3:** Results of chi-squared tests and logistic regressions for the primary endpoint.

Repetitions	Chi-Squared Test	*p*-Value	OR	Lower CI	Upper CI	*p*-Value
1	1.1	0.288	2.2	0.5	9.0	0.293
2	0.1	0.705	1.3	0.3	5.9	0.705
3	2.8	0.091	3.3	0.8	13.4	0.098
4	2.8	0.091	3.3	0.8	13.4	0.098
5	2.8	0.091	3.3	0.8	13.4	0.098
6	0.1	0.705	1.3	0.3	5.9	0.705
7	2.1	0.144	3.1	0.7	14.1	0.154
8	1.3	0.256	2.4	0.5	11.5	0.264
9	3.1	0.077	3.8	0.8	17.3	0.086
10	4.3	**0.038**	4.6	1.0	21.0	**0.047**
11	1.1	0.288	2.2	0.5	9.0	0.293
12	1.1	0.288	2.2	0.5	9.0	0.293
13	3.1	0.077	3.8	0.8	17.3	0.086
14	0.5	0.465	1.7	0.4	7.3	0.468
15	0.1	0.705	1.3	0.3	5.9	0.705
16	1.9	0.168	2.7	0.6	11.0	0.174
17	0.5	0.465	1.7	0.4	7.3	0.468
18	1.1	0.288	2.2	0.5	9.0	0.293
19	0.6	0.429	1.9	0.4	9.3	0.433
20	5.6	**0.018**	5.7	1.3	25.6	**0.024**
21	2.1	0.144	3.1	0.7	14.1	0.154
22	2.8	0.091	3.3	0.8	13.4	0.098
23	1.1	0.288	2.2	0.5	9.0	0.293
24	4.0	0.047	4.0	1.0	16.3	0.053
25	0.6	0.429	1.9	0.4	9.3	0.433

OR: odds ratio; CI: confidence interval. The bold numbers indicate *p* values < 0.05.

## Data Availability

The data presented in this study are available on request from the corresponding author.

## Supplementary Materials

The following supporting information can be downloaded at: https://www.mdpi.com/article/10.3390/jcm14010076/s1. Figure S1: Median chi-square convergence; the graph shows the difference between median chi-square with n repetitions and the median chi-square with n-1 repetitions; after 25 repetitions, the difference is null. Supplementary Table S1: Patients’ characteristics after PSM (Repetition 2); Table S2: patients’ characteristics after PSM (Repetition 20).
