# Supplementary material for: Cangrelor in Patients Undergoing Percutaneous Coronary Intervention After Out-of-Hospital Cardiac Arrest
Source: J Clin Med. 2024 Dec 27;14(1):76. doi: 10.3390/jcm14010076 (PMC11722389; doi:10.3390/jcm14010076)
Supplement: Supplementary file 1 [file jcm-14-00076-s001.zip › jcm-3337564-supplementary.pdf]

Supplemental Appendix

Supplemental Figure S1…………………..…………………………………………………………….1

Supplemental Table S1....…………...….……………………………………………………...…….2

Supplemental Table S2.....……………...……………………………………………….....……….3

Supplementary Figure S1. Median chi-squared test convergence

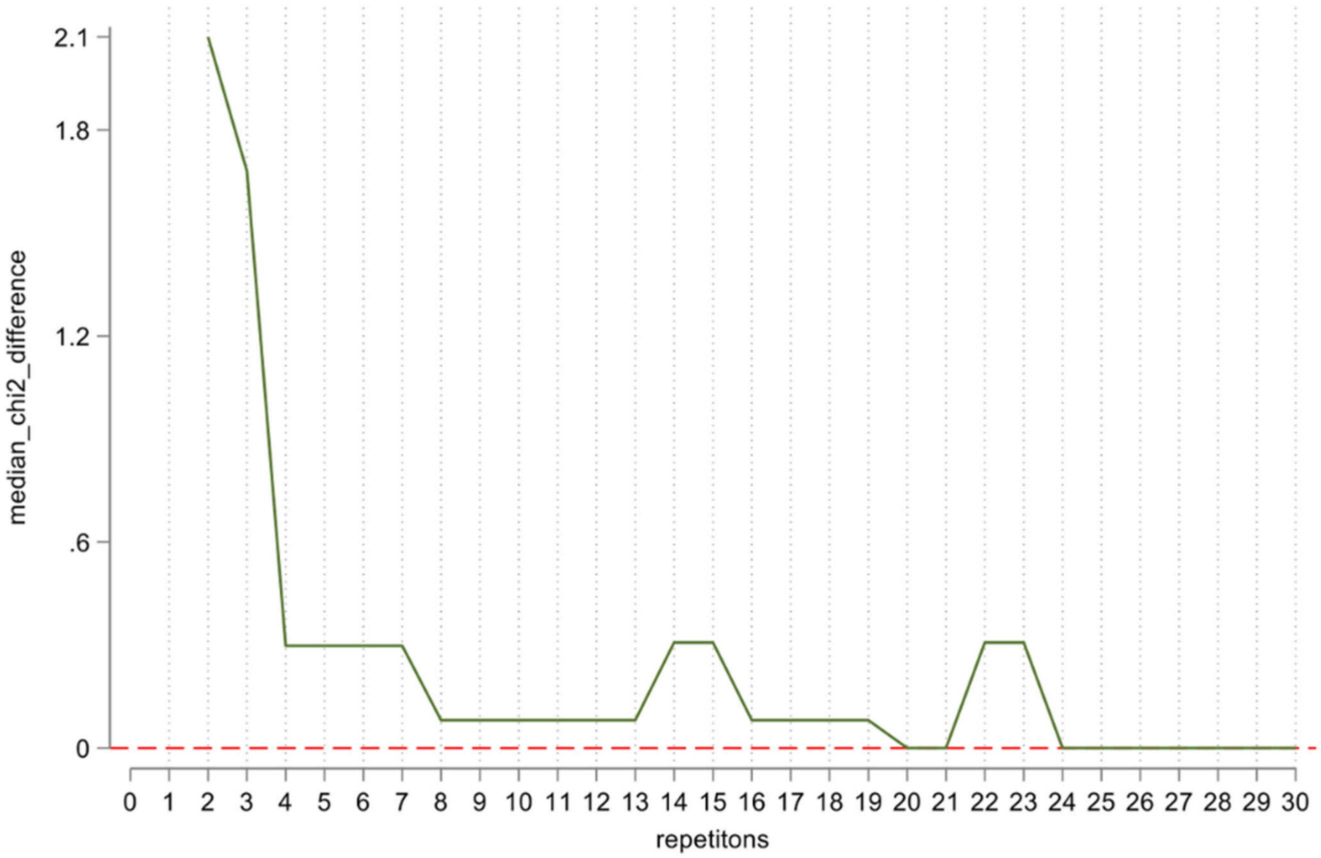

Graph showing the convergence of the median chi-squared test during propensity score matching. By repeatedly generating random samples based on the propensity score, the median chi-squared statistic is calculated across iterations. The y-axis represents the difference between the median chi-squared values for n repetitions and n-1 repetitions. Convergence is achieved when the difference between successive medians approaches zero, as seen after 25 repetitions.

Supplementary Table S1. Patients Characteristics After PSM (Repetition 2)

Clinical characteristics	No Cangrelor N=20	Cangrelor N=20	Overall N=40	p Value
Age (years)	63.5 (54.0-70.5)	61.0 (56.5-66.0)	62.0 (56.5-68.0)	0.38
Male	16 (80.0%)	16 (80.0%)	32 (80.0%)	1.00
BMI (kg/m^2)	25.8 (24.1-29.9)	24.7 (24.2-27.8)	25.7 (24.2-29.4)	0.58
Hypertension	17 (85.0%)	14 (70.0%)	31 (77.5%)	0.41
Diabetes	4 (20.0%)	3 (15.0%)	7 (17.5%)	0.56
Hypercholesterolemia	9 (45.0%)	7 (35.0%)	16 (40.0%)	0.52
Smoke	4 (20.0%)	9 (45.0%)	13 (32.5%)	0.05
Previous AMI	0 (0.0%)	1 (5.0%)	1 (725%)	0.35
High Bleeding Risk*	8 (40.0%)	2 (10.0%)	10 (25.0%)	0.11
PRECISE-DAPT score (n)	22.0 (10.0-40.0)	13.0 (7.5-21.5)	19.0 (9.0-33.0)	0.07
Peak high sensitivity Troponin (ng/L)	7795.0 (1429.0-47102.0)	57836.0 (7488.0-106185.5)	26602.5 (2005.5-85084.0)	0.12
ECG diagnostic for STEMI	9 (45.0%)	9 (45.0%)	18 (45.0%)	0.68
**Procedural characteristics**				
Femoral-access site for PCI (n)	9 (45.0%)	7 (35.0%)	16 (40.0%)	0.81
Pharmacological or mechanical hemodynamic support (n)	15 (75.0%)	15 (75.0%)	30 (75.0%)	1.00
Number of stents implanted (n)	1.0 (0.5-2.0)	1.0 (1.0-2.0)	1.0 (1.0-2.0)	0.63
**Procedural characteristics**				
Total length of stent implanted (mm)	38.0 (28.0-61.0)	33.0 (24.0-46.0)	36.0 (26.0-48.0)	0.54
LM PCI (n)	4 (20.0%)	3 (15.0%)	7 (17.5%)	0.68
GP IIb/IIIa receptor inhibitors used during PCI	11 (55.0%)	1 (5.0%)	12 (30.0%)	**0.001**
P2Y12 receptor inhibitors used during PCI				0.21
Clopidogrel	4 (20.0%)	6 (30.0%)	10 (25.0%)	
Ticagrelor	12 (60.0%)	14 (70.0%)	26 (65.0%)	
Duration of cangrelor infusion (h)		2.0 (2.0-2.5)		
Final TIMI flow 3 (n)	17 (85.0%)	19 (95.0%)	36 (90.0%)	0.25
**OHCA characteristics**				
Shockable rhythm at presentation	16 (80.0%)	19 (95.0%)	35 (87.5%)	0.15
Cardiac arrest time (min)	25.0 (20.3-47.0)	19.6 (4.0-29.9)	22.4 (12.0-42.0)	0.33
N° of delivered shocks (n)	1.5 (1.0-4.0)	2.0 (1.0-3.0)	2.0 (1.0-4.0)	0.87

This table corresponds to the repetition with the lowest OR observed for the primary endpoint (OR: 1.33; 95% CI: 0.30-5.93).

Supplementary Table S2. Patients Characteristics After PSM (Repetition 20)

Clinical characteristics	No Cangrelor N=20	Cangrelor N=20	Overall N=40	p Value
Age (years)	64.0 (54.0-70.5)	61.0 (56.5-66.0)	62.0 (55.0-69.5)	0.59
Male	16 (80.0%)	16 (80.0%)	32 (80.0%)	1.00
BMI (kg/m^2)	27.7 (25.1-30.2)	24.7 (24.2-27.8)	26.2 (24.2-29.4)	0.09
Hypertension	16 (80.0%)	14 (70.0%)	30 (75.0%)	0.54
Diabetes	4 (20.0%)	3 (15.0%)	7 (17.5%)	0.56
Hypercholesterolemia	8 (40.0%)	7 (35.0%)	15 (37.5%)	0.59
Smoke	9 (45.0%)	9 (45.0%)	18 (45.0%)	0.14
Previous AMI	2 (10.0%)	1 (5.0%)	3 (7.5%)	0.51
High Bleeding Risk*	7 (35.0%)	2 (10.0%)	9 (22.5%)	0.06
PRECISE-DAPT score (n)	30.5 (10.0-40.0)	13.0 (7.5-21.5)	20.0 (8.5-37.0)	0.06
Peak high sensitivity Troponin (ng/L)	36059.5 (2991.5-52200.5)	57836.0 (7488.0-106185.5)	36748.0 (4309.0-85084.0)	0.30
ECG diagnostic for STEMI	5 (25.0%)	9 (45.0%)	14 (35.0%)	0.39
**Procedural characteristics**				
Femoral-access site for PCI (n)	6 (30.0%)	7 (35.0%)	13 (32.5%)	0.54
Pharmacological or mechanical hemodynamic support (n)	13 (65.0%	15 (75.0%)	28 (70.0%)	0.49
Number of stents implanted (n)	1.0 (1.0-2.0)	1.0 (1.0-2.0)	1.0 (1.0-2.0)	0.42
**Procedural characteristics**				
Total length of stent implanted (mm)	29.0 (24.0-54.5)	33.0 (24.0-46-0)	30.0 (24.0-48.0)	0.78
LM PCI (n)	3 (15.0%)	3 (15.0%)	6 (15.0%)	1.00
GP IIb/IIIa receptor inhibitors used during PCI	12 (60.0%)	1 (5.0%)	13 (32.5%)	**0.001**
P2Y12 receptor inhibitors used during PCI				0.21
Clopidogrel	4 (20.0%)	6 (30.0%)	10 (25.0%)	
Ticagrelor	12 (60.0%)	14 (70.0%)	26 (65.0%)	
Duration of cangrelor infusion (h)		2.0 (2.0-2.5)		
Final TIMI flow 3 (n)	19 (95.0%)	19 (95.0%)	38 (95.0%)	0.37
**OHCA characteristics**				
Shockable rhythm at presentation	14 (70.0%)	19 (95.0%)	33 (82.5%)	0.05
Cardiac arrest time (min)	24.4 (10.0-36.2)	19.6 (4.0-29.9)	21.5 (5.0-31.0)	0.46
N° of delivered shocks (n)	1.0 (0.5-3.5)	2.0 (1.0-3.0)	1.5 (1.0-3.0)	0.25

This table corresponds to the repetition with the highest OR observed for the primary endpoint. (OR: 5.67; 95% CI: 1.25 -2 5.61)
